# Genetic and Lineage Classification of Glioma-Initiating Cells Identifies a Clinically Relevant Glioblastoma Model

**DOI:** 10.3390/cancers11101564

**Published:** 2019-10-15

**Authors:** Norihiko Saito, Nozomi Hirai, Kazuya Aoki, Sho Sato, Ryo Suzuki, Yu Hiramoto, Satoshi Fujita, Haruo Nakayama, Morito Hayashi, Takatoshi Sakurai, Satoshi Iwabuchi

**Affiliations:** Department of Neurosurgery, Toho University Ohashi Medical Center, Tokyo 153-8515, Japan; nozomi.hirai@med.toho-u.ac.jp (N.H.); kaoki@med.toho-u.ac.jp (K.A.); sho.sato@med.toho-u.ac.jp (S.S.); ryo.suzuki@med.toho-u.ac.jp (R.S.); yu.hiramoto@med.toho-u.ac.jp (Y.H.); satoshi.fujita@med.toho-u.ac.jp (S.F.); haruonakayama@med.toho-u.ac.jp (H.N.); morito@med.toho-u.ac.jp (M.H.); cherry@med.toho-u.ac.jp (T.S.); iwabuchi@med.toho-u.ac.jp (S.I.)

**Keywords:** glioblastoma, glioma initiating cell, The Cancer Genome Atlas, molecular classification

## Abstract

The Cancer Genome Atlas (TCGA) project described a robust gene expression-based molecular classification of glioblastoma (GBM), but the functional and biological significance of the subclasses has not been determined. The present comprehensive analysis of 25 glioma-initiating cell (GIC) lines classifies GIC lines into four subtypes (classical, mesenchymal, proneural, and neural) that are closely related to the TCGA GBM subclasses and display distinct lineage characteristics and differentiation behavior that recapitulate neural development. More importantly, the GIC subtypes exhibit distinct biological phenotypes in relation to self-renewal capacity, proliferation, invasiveness, and angiogenic potential in vitro and in vivo. In addition, the GIC subtypes exhibit divergent patterns of signaling pathway activation and deactivation of the Wnt, Notch, and TGF-β pathways. These results will improve drug discovery targeting certain genetic mutation in glioblastoma and improve the development of precision medicine.

## 1. Introduction

Despite advances in our understanding of the molecular aspects of glioblastoma (GBM), the prognosis for glioblastoma remains dismal [1,2,3,4]. Recent studies indicate that some neoplastic cells within human glioblastoma are capable of self-renewal and multilineage differentiation—properties associated with normal neural stem cells [5,6,7]. These stem-like tumor cells, known as glioma-initiating cells (GICs), are responsible for tumor initiation and recurrence after therapy, which makes them attractive targets for novel GBM therapies [8,9,10]. Other studies indicate that GICs retain relevant molecular features of human GBM and therefore may enable development of better preclinical models for evaluating tumor biology and therapeutics [11].

GICs cultured under neural stem cell conditions exhibit heterogeneous biological characteristics and molecular profiles. Previous studies classified GICs based on CD133 expression and found that the CD133-negative subtype had a lower proliferation index [12]. Gunther et al. [13] proposed that two major GIC subtypes exist. One exhibits a full stem-like phenotype, with spherical growth in vitro, expression of CD133, capacity for broad neuroglial differentiation, and high tumorigenicity and invasive growth in vivo. The other displays a restricted stem-like phenotype that only partially satisfies the relevant criteria. More recent work from Lottaz et al. [14] divided 17 GICs into two groups, one expressing a “proneural” gene signature resembling normal fetal brain stem cells and the other expressing a mesenchymal gene signature, which is more similar to that of adult neural stem cells. These findings suggest that differences in GBM tumors may be related to variation in GIC ancestors. 

Several signaling pathways are involved in maintaining stemness and tumorigenicity of GICs. The TGF-β pathway is active in high-grade gliomas and is associated with a poor prognosis [15,16]. It increases the self-renewal capacity of GICs through the induction of Leukemia inhibitory factor (LIF) and Sex determining region Y-box 2 (Sox2) and subsequent activation of the JAK-STAT pathway [7,17]. Notch signaling is required in order to prevent neuronal differentiation and promote neural stem cell maintenance for further commitment into a glial lineage [18]. In mice, blockade of Notch signaling reduced GIC growth and clonogenicity in vitro, as well as tumor formation [10,19]. Other pathways involved in GIC maintenance and function include the basic fibroblast growth factor (bFGF), Wnt, and the hedgehog signaling pathways [20,21,22]. Signal transduction pathways that modulate GIC growth and differentiation have been extensively studied; however, it remains unclear if these signals equally or differentially contribute to stem cell maintenance in GICs.

A distinguishing feature of GBM is marked genetic heterogeneity within individual tumors. Phillips and colleagues described a 35-gene signature that divided GBM into three subtypes (which resembled different stages of neurogenesis) that correlated with prognosis and established a pattern of disease progression [23]. Recently, The Cancer Genome Atlas (TCGA) included a robust gene expression-based molecular classification of GBM into proneural, neural, classical, and mesenchymal subtypes and highlighted strong relationships with the different neural lineages [24]. Further studies are needed in order to interpret the biological and functional significance of these GBM subclasses.

In this study, we analyzed the gene expression profiles of 25 GICs derived from fresh GBM tissues and identified four GIC subtypes, which closely resemble the recently identified TCGA GBM subclasses. We also observed diverse lineage characteristics in the GIC subtypes and differentiation behavior recapitulating neural development. In addition, the GIC subtypes exhibited distinct biological phenotypes in relation to self-renewal capacity, proliferation, invasiveness, and angiogenic potential in vitro and in vivo. More importantly, the GIC subtypes exhibited divergent patterns of signaling pathway activation and deactivation of the Wnt, Notch, and TGF-β pathways. This comprehensive genetics-based classification of GICs lays the groundwork for a better molecular understanding of GBM pathway signaling, which could assist in the development of personalized therapies for certain GBM patient subgroups.

## 2. Results

### 2.1. Identification of Four GIC Subtypes by Cluster Analysis of Gene Expression Profiling Data

**Figure 1:**
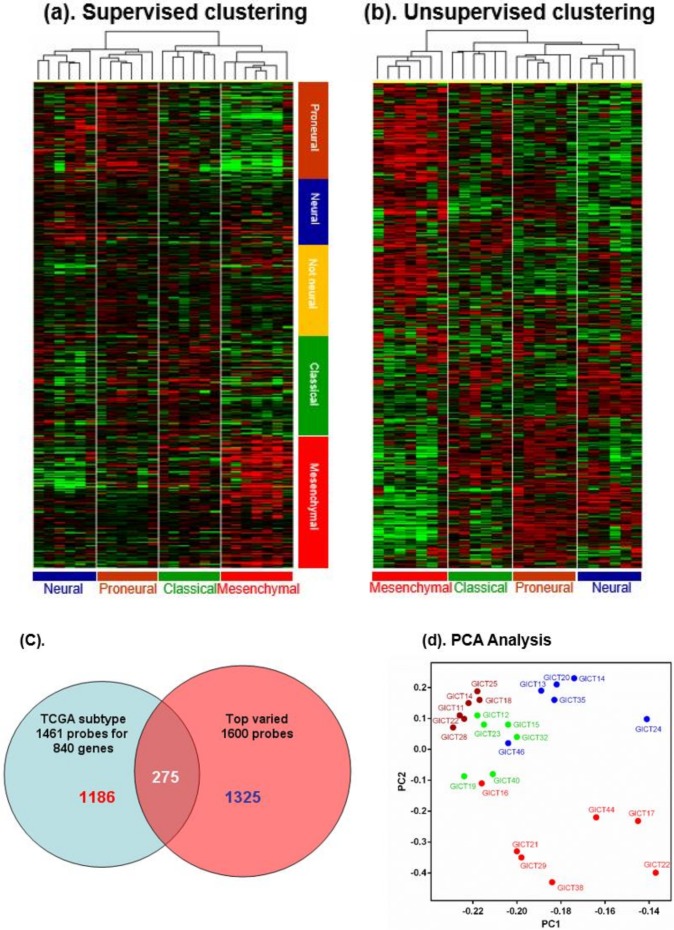
(**a**,**b**): Supervised clustering (The Cancer Genome Atlas (TCGA) 1461 probe sets) and unsupervised clustering (MTA 1600 probe sets) classified 25 glioma-initiating cell (GIC) cell lines into mesenchymal, neural, proneural, and classical subtypes. Defined GIC subtypes could be distinguished by their distinct patterns of gene expression, as related to TCGA subclass. (**c**) There were 275 probe sets (~20% of total) common to these two methods. (**d**) PCAof gene expression of the four GIC subtypes indicated that the proneural and classical GICs were most closely related and that neural and mesenchymal GICs deviated in a direction opposite to them, which supports the hypothesis that the “proneural” group has two GIC subtypes.

To identify potential GIC subtypes, we performed cluster analysis of gene expression data (Affymetrix U133A2) from 25 GIC cell lines generated from fresh GBM tissues (Table S1). Two approaches were used for the analysis. First, we performed supervised clustering using 1461 probe sets matching 840 genes reported to be differentially expressed in GBM subclasses, which effectively separated GBMs into four previously described subclasses [24]. This separated 25 GICs into two major clusters-one comprising subclusters of GICs with high expressions of “neural” and “proneural” genes and another comprising subclusters of GICs with high expressions of “classical” and “mesenchymal” genes (Figure 1a). Second, we performed unsupervised clustering with the top 1600 varied probe sets. Surprisingly, this yielded four subclusters that exactly matched those identified in supervised clustering (Figure 1b). There were 275 probe sets (~20% of total) common to these two methods (Figure 1c). This second method was better able to separate “mesenchymal” genes, presumably because more “mesenchymal” gene probe sets were present in this clustering. Notably, the “proneural” subclass showed expression of “proneural” and “classical” subtypes. The fact that both methods yielded the same classification pattern confirmed our clustering results and further indicates that subtypes specific to GBM gene expressions might be determined by varied GIC subtypes that drive proliferation and progression of these tumors. Some genes highly enriched in GIC subtypes were bone morphogenetic protein 4 (BMP4), doublecortin (DCX), p16INK4a, and inhibitor of differentiation 2 (ID2) for the neural subtype; oligodendrocyte lineage transcription factor 2 (OLIG2), NK2 homeobox 2 (NKX2-2), Notch1, and Notch3 for the proneural and classical subtypes; and transforming growth factor beta receptor 2 (TGFBR2), CD44, and caveolin 1 (CAV1) for the mesenchymal subtype. Principal component analysis (PCA) of gene expression of the four GIC subtypes (Figure 1d) indicated that the proneural and classical GICs were most closely related and that neural and mesenchymal GICs deviated in a direction opposite to them, which supports the hypothesis that the “proneural” group has two GIC subtypes.

### 2.2. Distribution of Frequently Mutated Genes Across GIC Subtype Is Relevant to TCGA GBM Subclass

Mutation analysis was performed for a panel of genes known to be important in GBM. The p53 mutation was enriched in neural and mesenchymal subtypes, whereas the phosphatase and tensin homolog (PTEN) mutation was prevalent in classical and mesenchymal GICs. Mutation of *p53* and *IDH1* was present in neural GICs, whereas PI3K-kinase mutations (PI3CA) were present only in proneural GICs. In addition, co-mutation of *p53, IDH1*, and *Akt 3* was observed in the neural subtype; however, co-mutation of *PI3K* genes was rare in all subtypes (Table 1). No EGFR mutation was present in GICs.

### 2.3. GIC Subtypes Exhibit Distinct Lineage Characteristics That Recapitulate Neural Development

**Figure 2:**
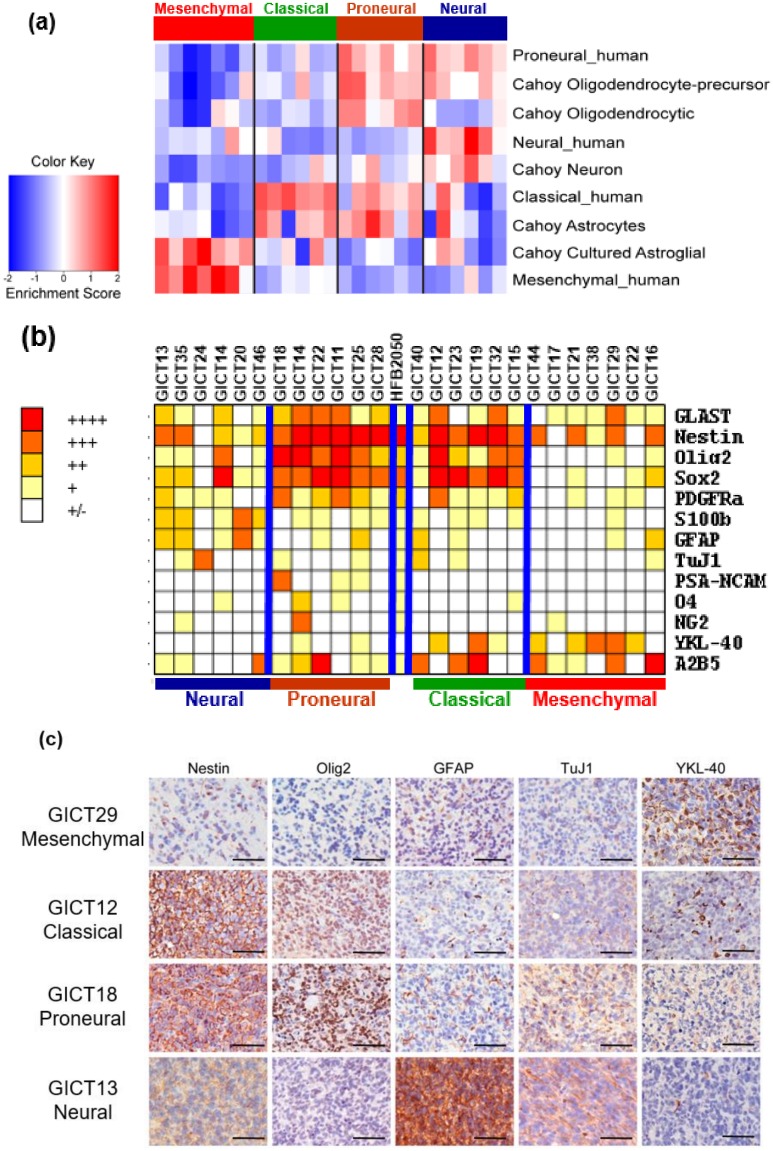
(**a**) GIC subtypes differentially expressed lineage markers for neural stem/progenitor cells (neuroepithelial cells, radial glia, glial progenitors, neuronal progenitors, oligodendrocyte progenitor cells, and SVZ astrocytes). (**b**) GIC subtypes exhibited distinct lineage profiles that recapitulated neural development. Earlier lineage markers (Nestin, Sox2, Olig2) are highly expressed in proneural and classical GICs, while differentiation markers (GFAP: glial fibrillary acidic protein, TuJ1: Neuron-specific beta-III Tubulin) are abundant in neural GICs. YKL-40: Chitinase-3-like protein 1 is enriched in mesenchymal GICs. (**c**): Lineage characteristics of GIC subtypes are retained in their intracranial xenografts. One of representative results of each subtypes are illustrated in Figure 2C. Scale bars: 100 μm.

To clarify the biological importance of the subtypes, we used probe set data presented by Cahoy et al. [25] to define gene sets associated with neurons, oligodendrocytes, astrocytes, and cultured astroglial cells. These four gene sets were used to calculate a single-sample gene set–enrichment analysis (ssGSEA) enrichment score for all samples, to indicate how closely expression in a sample reflects the expected expression pattern of the gene set. This analysis yielded a number of patterns that associated each subtype with expression patterns from purified murine neural cell types (Figure 2a). The proneural subtype was highly enriched with proneural, oligodendrocytic, and astrocytic signatures, whereas the classical group was exclusively associated with the murine astrocytic signature. Neural subtype was associated with proneural signature but also showed strong enrichment for genes differentially expressed by neurons. This suggests that the neural subtype is more committed than the proneural subtype in differentiated lineages. The mesenchymal subtype was exclusively associated with the in vitro cultured astroglial signature. These results are highly consistent with the lineage profiles of TCGA GBM subclasses [24].

Immunostaining was used to examine expressions of a panel of neural lineage markers in GIC subtypes (Figure 2b). GICs differentially expressed lineage markers related to specific classes of neural stem/progenitors and exhibited a bidirectional tendency toward neural or mesenchymal development. Notably, expressions of neural stem cell markers Sox2, Olig2, and Nestin were higher in proneural and classical subtypes, whereas expressions of glial fibrillary acidic protein (GFAP) and S100 calcium binding protein B (S100β), an astrocytic marker, were higher in neural GICs. Glial progenitor markers such as A2B5 and glutamate aspartate transporter (GLAST) were also expressed in proneural and classical subtypes. Mesenchymal GICs highly expressed the mesenchymal marker YKL-40, but positivity on neural lineage markers was limited. These findings suggest that classical and proneural GICs are more similar to the normal human fetal brain–derived stem cell line HFB2050. Further, classical and proneural subtypes are closely related and are a more primitive stem cell, whereas the neural subtype includes cells that are more differentiated. We also compared marker expression pattern in in vitro culture and xenografts in relation to GIC subtype (Figure 2c). Our results showed that GIC subtype lineage characteristics were retained in the xenograft: classical and proneural xenografts highly expressed Nestin, Olig2, and YKL40, and the mesenchymal xenograft had high YKL-40 expression but low expressions of Nestin, GFAP, and Olig2.

### 2.4. GIC Subtypes Exhibited Distinct Differentiation Behavior In Vitro

**Figure 3:**
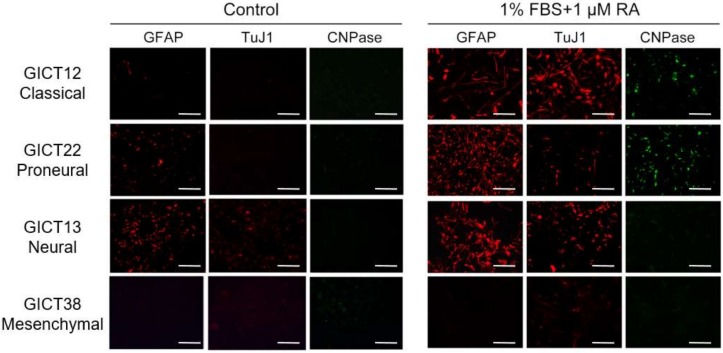
GIC subtypes cultured under different conditions (1% (fetal bovine serum) FBS + 1 µM RA) exhibited varied potential for differentiation into neural lineages. Most proneural and classical GICs displayed trilineage differentiation potential, while neural GICs rarely differentiated into the oligodendrocytic lineage. Mesenchymal GICs were less likely to differentiate into astrocytic, neuronal, and oligodendrocytic lineages. One of representative results of each subtypes are illustrated in Figure 3. Scale bars: 100 μm.

The differentiation potential of GIC subtypes was examined in culture under 1% (fetal bovine serum) FBS plus 1 μM retinoic acid. Classical and proneural GICs were able to differentiate into astrocytic, neuronal, and oligodendrocytic lineages. While neural GICs were mainly able to differentiate into astrocytic and neuronal lineages, mesenchymal GICs seemed resistant to FBS and retinoic acid (RA) treatment and failed to differentiate into neural lineage cells (Figure 3, Table S2).

### 2.5. Differential Growth Characteristics in GIC Subtypes

**Figure 4:**
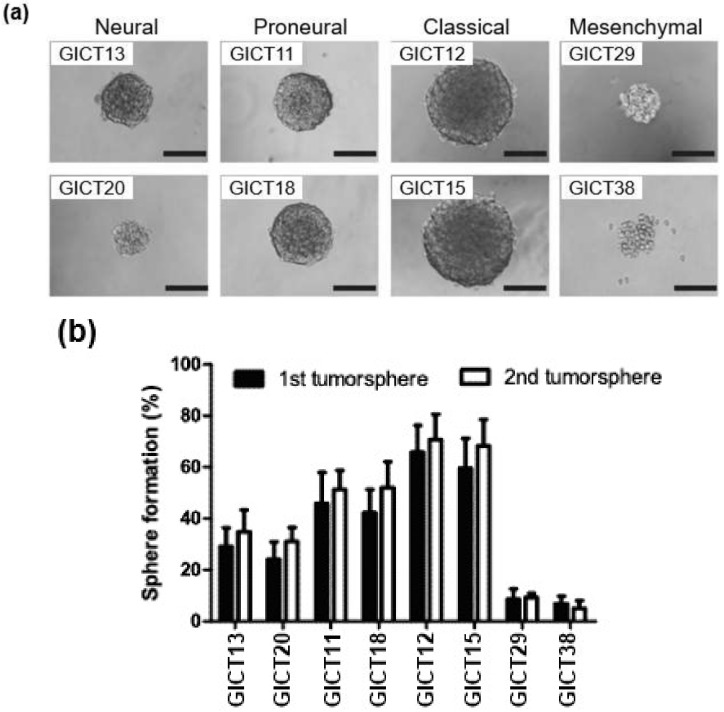
(**a**,**b**): GIC subtypes exhibit distinct biological behaviors in relation to self-renewal capacity. Representative results of each subtypes are illustrated in Figure 4a,b. Scale bars: 250 μm.

To identify growth characteristics of GIC subtypes and evaluate their capacity to form tumor spheres, we examined their growth properties in vitro. The GIC subtypes exhibited varied potential to form primary and secondary tumor spheres (Figure 4a,b). While classical GICs readily formed large tumor spheres, mesenchymal GICs did not. Instead, they formed loosely packed tumor spheres that were smaller than those of other subtypes.

### 2.6. GIC Subtypes Exhibit Varied Biological Behavior In Vitro and In Vivo

**Figure 5:**
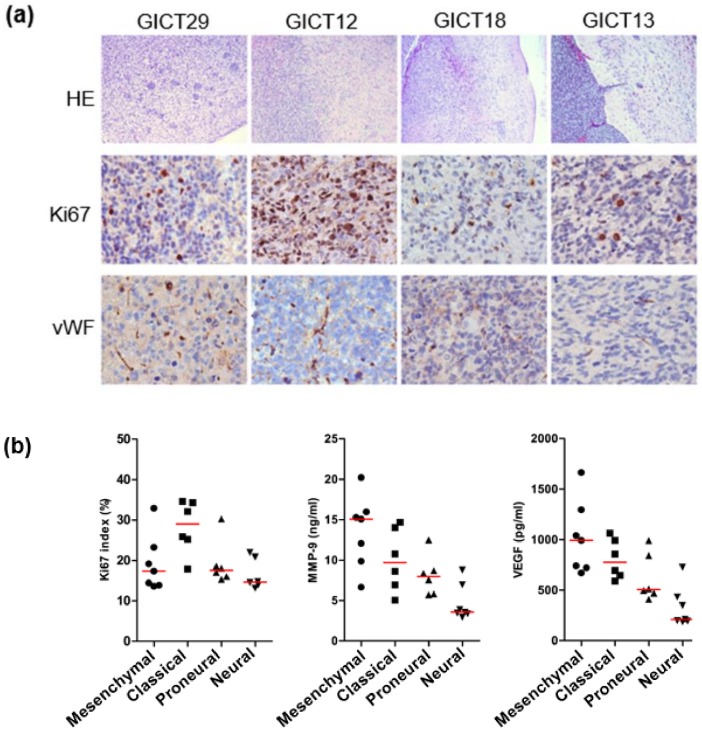
(**a**) The in vivo biological behavior of GIC subtypes was studied by orthotopic injection of cells into mouse brain. The neural xenograft subtype was the least angiogenic, as indicated by vWF staining. Ki67 staining showed that classical GICs were most active in proliferation in vivo. Scale bars: 100 μm. (**b**) Neural GICs produced significantly lower amounts of VEGF and MMP-9.

The in vivo biological behavior of GIC subtypes was studied by orthotopic injection of cells into mouse brain. The histological phenotypes of tumors formed differed in relation to GIC subtype (Table 2). Mesenchymal GICs exhibited high tumorgenicity in vivo despite the absence of CD133 expression. Mesenchymal xenografts were highly invasive, whereas neural xenografts were the least invasive, forming a well-defined tumor margin separating tumor from surrounding normal tissues (Figure 5a). In addition, the neural xenograft subtype was the least angiogenic, as indicated by von Willebrand factor(vWF) staining (Figure 5a). These results are consistent with those of in vitro culture, which showed that neural GICs produced significantly lower amounts of vascular endothelial growth factor (VEGF) and matrix metallopeptidase 9 (MMP-9) (Figure 5b)—key regulators of tumor angiogenesis and invasion, respectively. The proliferative potential of GICs was examined by ki67 staining in cultured GIC and xenografts (Figure 5a,b). Ki67 staining showed that classical GICs were most active in proliferation in vitro and in vivo; they exhibited intratumoral necrotic regions that were coextensive with extravasating blood cells. Thus, the in vitro and in vivo characteristics of GIC biological behaviors support the bioinformatic classification of GICs.

### 2.7. Signaling Pathway Activation and Deactivation Regulate GIC Subtype

**Figure 6:**
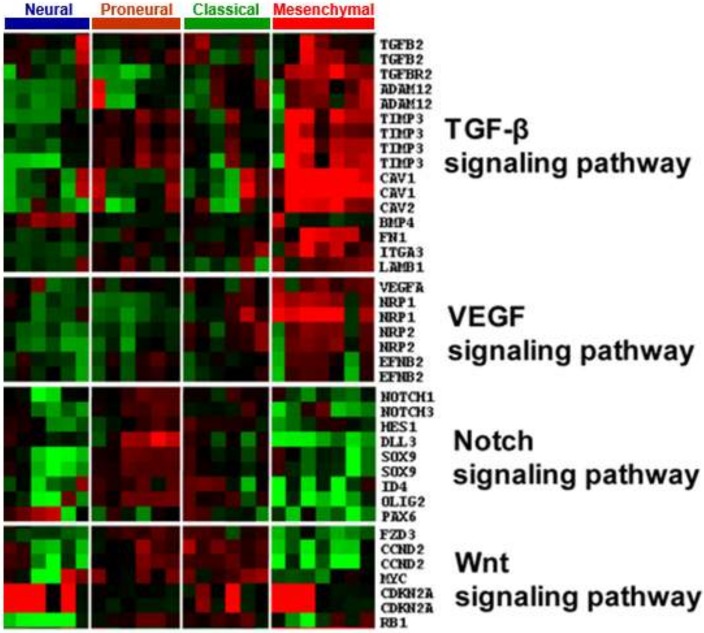
GIC subtypes exhibited divergent patterns of signaling pathway activation. Multiple pathways, such as TGF-β, Notch, VEGF, and Wnt, were identified in GIC subgroups.

GICs exhibited subtype-specific lineage markers and differentiation potentials, which is reminiscent of the hierarchical normal neural development process in which signaling pathways (e.g., the Notch and Wnt pathways) are differentially tuned. ANOVA analysis was used to identify genes that were significantly differentially expressed among GIC subtypes (*p* ≤ 0.05). The genes identified overlapped with a manually curated gene list comprising known genes involved in the Wnt, Notch, and TGF-β signaling pathways, which revealed differential activation and deactivation of signaling pathways within GIC subtypes. In mesenchymal GICs, the TGF-β pathway component was highly expressed, concurrent with deactivation of Notch and Wnt, as physiological inhibitors of Notch and Wnt are abundant in this subtype. Notch pathway components were highly enriched in classical and proneural GICs but deactivated in neural GICs (Figure 6).

## 3. Discussion

Accumulating evidence from TCGA has yielded a robust gene expression–based molecular classification of GBM into proneural, neural, classical, and mesenchymal subtypes [24]. Identification of valid GBM subtype counterparts in GICs represents an important aid to studying GBM subtypes, in particular for modeling and predicting therapeutic response. However, numerous important questions remain unanswered, such as the extent to which the GIC model preserves the genetic and biological features of GBMs during in vitro culture. Here, we report the establishment of GIC lines and identify four GIC subtypes by means of clustering analysis of gene expression profiles. Our results show that unsupervised clustering of GIC gene expression analysis matched perfectly with that from a supervised clustering using 840 genes previously used to separate GBM tumors into four subtypes. These findings confirm that, despite in vitro handling of GIC lines, the transcriptome of GIC lines resembles that of primary GBM tumors. Our study identified four subtypes (neural, proneural, classical, and mesenchymal) in GIC lines, though unsupervised clustering contained only ~20% of supervised clustering probe sets, which suggests that these subtypes are intrinsic within GIC lines and may be drivers of GBM subtypes.

Recent genome-wide profiling studies have reported genetic abnormalities associated with GBM subtypes [24,26]. These studies reported p53 gene mutations, mostly in proneural and mesenchymal GBM, and PTEN gene mutations in classical and mesenchymal GBM [11,24]. Our GIC study showed a similar distribution profile, although frequencies were higher, suggesting an intrinsic association between GBM and GIC. In addition, a higher frequency of mutations in PIK3CA and PIK3R1 was observed in GIC subtypes, but none coexisted in the same GIC line; thus; mutation in either *PI3K* gene may be sufficient to drive GIC growth. Although some studies found no *AKT1* mutation in GBM [27,28], we identified the *AKT3E17K* mutation in GIC. AKT3 has a pivotal role in human GBM biology [29]; therefore, assessing the functional role of AKT3 activation by somatic mutations in GBM is relevant in identifying its role in this aggressive disease. In addition, IDH1 is mutated in >80% of secondary GBM, although <10% of primary GBM harbor these alterations [30,31]. Recently, TCGA revealed IDH mutation in proneural GBM and frequent co-mutation with p53 [24]. Our data confirm the low frequency of IDH1 mutation in a GIC line derived from primary GBM and revealed co-mutation of IDH1 and p53 in a proneural GIC line, which suggests that co-mutation of IDH1 and p53 is crucial in maintaining this GIC subtype. An important finding of TCGA analysis was that EGFR gene mutation was present in almost half of GBM tumors examined. However, no EGFR mutation was seen in our GIC lines, suggesting that cells with mutated EGFR might be lost or selected against during culture.

The developing and adult nervous system has distinct classes of neural stem/progenitors in the lineage hierarchy. Recent studies reported that glioma cells expressing lineage markers such as A2B5 [32], NG2 [33], CD44 [29], and even GFAP also meet the criteria for tumorigenic stem cells, suggesting that GIC originate from a broader spectrum of neural lineages. Our findings indicate that GICs are a heterogeneous population and that classical and proneural subtypes are more primitive, that neural subtype is more differentiated, and that the mesenchymal subtype seems to deviate from neural lineage through mesenchymal transition expressing the mesenchymal marker YKL40. The lineage hierarchy status of GIC subtypes may confer variation in the tendency to differentiate; the proneural and classical GIC subtypes, for example, highly express markers of the stem/early progenitors and can differentiate into trilineage cells. By contrast, the neural subtype exhibits higher GFAP expression, like adult subventricular zone (SVZ) astrocytes, and has less potential upon induction to differentiate into bilineage cells. The mesenchymal subtype of GIC is almost incapable of differentiating into neural lineage cells. Thus, the fact that gliomas have a large, continuous histological spectrum with regard to proportions of various differentiated and anaplastic cells may be attributable to the phenotype of the underlying tumor-initiating cells. If combined with mutation analysis, it is plausible that neural stem/progenitors or even differentiated cells can be converted to a stem cell state through genetic disruptions of specific sets of genes.

Tumor stem cells reside in a microenvironment known as the “stem cell niche”, which maintains them in a stem-like state [34]. Multiple recent studies have clearly demonstrated that—by delivering special signals to balance cell proliferation, self-renewal, and differentiation-the stem cell niche within glioma is a key regulator of stem cell fate [35,36,37]. The present data show that Wnt, Notch, and TGF-β signals are prominent in controlling cell deactivation of proliferation, self-renewal, and differentiation in GICs. Whether these signals equally or differentially contribute to stem cell maintenance in GICs remains unresolved [10,11]. We found that GICs exhibited subtype-specific activation and deactivation of these signaling pathways. Interestingly, in the mesenchymal subtype, TGF-β signaling was concurrent with attenuation of Wnt and Notch signaling. In contrast, in the proneural subtype, Notch signaling, rather than TGF-β, was highly activated. Disruption of these signaling pathways by specific inhibitors impaired growth of GIC subtypes. These results suggest that GIC subtypes might be maintained by varied activation of signaling pathways. Therefore, targeting these GIC subtypes by utilizing potential differences in stem cell–niche interaction might allow successful stem cell targeting and improve outcomes of cancer treatment.

## 4. Materials and Methods

### 4.1. Cell Lines and Cell Culture

The 25 glioma initiating cell lines were maintained in neurosphere medium by using a previously described method [38] to isolate neurosphere-forming cells from surgical specimens of human GBM. The study was approved by the Institutional Review Board of Toho University (H22-62). These GIC lines were cultured as GBM neurospheres in DMEM/F12 medium supplemented with B27 (Invitrogen, Grand Island, NY, USA), L-glutamine (GIBCO), penicillin/streptomycin, and growth factors (20 ng/mL EGF and 20 ng/mL FGF-2; Invitrogen). The human fetal brain neural stem cell line HFB2050 was kindly provided by Evan Snyder and cultured as described previously. For differentiation analysis, GICs were grown on polylysine-coated coverslips and treated with retinoic acid (1µM) (Sigma-Aldrich, St. Louis, MO, USA) plus 1% fetal bovine serum (Sigma-Aldrich, St. Louis, MO, USA) in DMEN/F12 for 5 days.

### 4.2. Gene Arrays and Bioinformatic

GIC expression profiles were generated on an Affymetrix GeneChip HG-U133A 2.0 microarray platform [37]. Clustering analysis was performed with Cluster and Tree View software (Cluster 3.0, Software copyright Stanford University, USA). For supervised clustering, we used 1461 probe sets that matched 840 previously identified subtype-specific genes to cluster GIC samples. The parameters used were correlation uncentred and average linkage. For unsupervised clustering, we used the top 1600 varied probes among all GIC samples and clustered both on samples and probe sets. The parameters used were Spearman ranking and average linkage. PCA analysis of the top 1600 varied probes was done with the R software package, after background removal, and the first and second components were scatter-plotted. Single-sample gene set–enrichment analysis (ssGSEA) scores were calculated by using previously reported methods [24]. Briefly, genes were ordered by their expression values. The empirical cumulative distribution functions (ECDF) of genes in the signature and in the remaining genes were calculated. The enrichment score was defined as the sum of the difference between the weighted ECDF of the genes in the signature and the ECDF of the remaining genes. This calculation was reported for all signatures and samples. A positive score indicates a high ranking of up-genes and a low ranking of down-genes in the signature. A negative score does not indicate the opposite, but rather lack of effect. Gene sets for four GBM expression subtypes were obtained from Verhaak et al. [24]. The gene signatures for six different neural lineages were obtained from Cahoy et al. [27]. Lineage cell-related gene sets were generated by using the transcriptome database presented in Cahoy et al. [25]. A positive enrichment score indicates a positive correlation between genes in the gene set and the expression of profile tumor samples; a negative enrichment score indicates an inverse correlation.

### 4.3. Somatic Mutation Analysis

Genomic DNA from all GIC samples was purified and subjected to phi29 polymerase multiple strand-displacement whole-genome amplification with the Sequeon OncoCarta Panel [39]. All analyses were performed in duplicate and repeated to measure assay reproducibility. Exon sequencing was done for the *p53* and *PTEN* genes. We selected only nonsynonymous coding mutations that were previously reported as somatic mutations in human cancer, as described in the Cosmic, PubMed, and TCGA datasets.

### 4.4. Immunofluorescence Staining

Immunofluorescence staining was performed as described previously [40]. Cells were seeded at a concentration of 2 × 10^5^ cells/well in six-well plates with polylysine-coated coverslips inside and left for 24 h in the incubator. The cells were washed once with phosphate-buffered saline (PBS) (Sigma-Aldrich, St. Louis, MO, USA) before being fixed with 4% formaldehyde in PBS for 20 min. After another PBS wash, the cells were permeabilized with 0.1% Triton X-100 in PBS for 5 minutes and then blocked with 5% goat serum in PBS (containing 0.1% Tween 20) for 1 h. Cells were then incubated with primary antibodies (anti-Nestin and anti-GFAP, anti–YKL-40 from Cell Signaling, anti-CNPase from Millipore, anti-TuJ-1 from Sigma-Aldrich, and anti-Olig2 from Abcam) overnight at 4 °C. After 2 washes with PBS (containing 0.1% Tween 20), the cells were incubated with the indicated fluorescently labeled secondary antibody in darkness at room temperature for 1 h. The cells were counterstained with Vectashield sealant containing 4′,6-diamidino-2-phenylindole (DAPI) (Vector Laboratories, CA, USA) and examined under a confocal laser scanning microscope (Carl Zeiss Microscopy, Germany). Images were scanned with a DFC340FX camera (Leica Microsystems, Germany). The percentage of positively stained cells in each section was determined at a magnification of 400× by counting 500 cells in a randomly selected field.

### 4.5. Immunohistochemical Staining

Sections (thickness 5 μm) of formalin-fixed, paraffin-embedded whole brain from animal specimens were stained with the primary antibodies listed in Table 3. The sections were visualized by using a diaminobenzidine substrate kit. The slides were examined under a bright-field microscope [10].

### 4.6. ELISA

We used a human VEGF enzyme-linked immunosorbent assay (ELISA) to quantify secretory VEGF and MMP-9 in conditioned media, in accordance with the manufacturer’s instructions (R&D Systems, Minneapolis, MN, USA). For analysis of MMP-9 and VEGF, 1 × 10^5^ GICs were plated in a 12-well plate for 48 h, and VEGF was quantified in the conditioned media, as described previously. Data analysis was performed with Microplate Manager Software (Molecular Device, BioRad, Hercules, CA, USA). The VEGF/MMP-9 concentration in the samples was calculated by interpolation from the standard curve. Total protein was determined, to normalize ELISA.

### 4.7. Neurosphere Formation Assay

For the primary neurosphere formation assay, GICs were seeded in 96-well plates (100 cells/well) and incubated for 7 days. For the secondary neurosphere formation assay, primary neurospheres were dissociated into single cells, 50 of which were seeded in each well of 96-well plates in the absence of inhibitors. After 7 days, total neurosphere numbers were counted with a dissection microscope using 4 × 4 objective magnification.

### 4.8. Animal Models

The mice were housed and cared for at the animal care facility of Toho University School of Medicine in accordance with the institution’s guidelines for the care and use of laboratory animals. The experimental protocol was approved by the Animal Research Committee, Toho University School of Medicine (ARC/TUSM-R16-14). To create the intracranial xenograft model, we engrafted GICs into the caudate nucleus of each mouse, using a previously described guide-screw system [10]. Briefly, 5 × 10^5^ dissociated tumor cells in DMEM/F-12 serum-free media (5 µM) were injected stereotactically into Nude (nu/nu) 6–8-week-old mice. Mice were monitored daily and euthanized when they became moribund. At necropsy, all organs were analyzed grossly and microscopically.

### 4.9. Statistical Analysis

Statistical analysis was performed by using the Student unpaired t-test. Results are presented as the mean of at least three independent experiments. Survival analysis was performed by using the log-rank analysis module in SPSS 10.0 (SPSS Inc, Chicago, IL, USA). Differences were considered significant at *p* < 0.05, for all comparisons.

## 5. Conclusions

In conclusion, the present comprehensive analysis revealed distinct GIC subtypes that are closely related to TCGA GBM. They appear to derive from different cells of the neural lineage through varied molecular processes, such as gene mutation and aberrant signaling activation. Importantly, the lineage profiles and biological phenotypes of GIC subtypes were retained by in vivo xenografts. These findings improve our understanding of GBM development and the heterogeneity of these tumors and may hasten development of more personalized and improved therapies targeting GICs. Thus, GIC is biologically and molecularly a more relevant model system for preclinical studies of therapeutic intervention and for improving our understanding of the molecular biology of human GBM.

## Figures and Tables

**Table 1:** Mutation analysis in GICs.

Subtype	Cell Line	TP53	PTEN	PIK3CA	PIK3R1	MET	IDH1	N-Ras	AKT3
Neural	GICT14	S241Y					R132H		E17K
	GICT12	G105D	Del exons 2–9						
	GICT46		Y88 *						
	GICT20	P98L							
	GICT24	R306 stopcodon							
	GICT35	G105D	Del exons 2–9						
Proneural	GICT11			Q546P					
	GICT22			Q546P					
	GICT14			R88Q					
	GICT25	R273C							
	GICT18		Exon 6Del AGAA						
	GICT28		Y68H		M326I				
Classical	GICT15	M237I	Del exons 1						
	GICT12		Del exons 1–2		M326I				
	GICT32				M326I	R988C			
	GICT23		R233 *		N564K				
	GICT40	C238Y				N375S			
	GICT19		Deletion all exons						
Mesenchymal	GICT17	H179R	M199del					Q61K	
	GICT38		Del exons 3–9						
	GICT29	C238Y				N375S			
	GICT44	P98L							
	GICT22		R233 *						
	GICT16		G132D						
	GICT21		Del exons 2–9						

TP53: tumor protein p53; PTEN: phosphatase and tensin homolog; PIK3CA: phosphatidylinositol-4,5-bisphosphate 3-kinase catalytic subunit alpha PIK3R1: phosphoinositide-3-kinase regulatory subunit 1; MET: MET proto-oncogene, receptor tyrosine kinase; IDH1: isocitrate dehydrogenase 1; N-Ras: NRAS proto-oncogene; AKT3: AKT serine/threonine kinase 3; *:Nonsense mutation.

**Table 2:** The histological phenotypes of tumors formed differed in relation to GIC subtype.

Histological Phenotype	Neural	Proneural	Classical	Mesenchymal
In vivo invasion	Less invasive	invasive	invasive	Highly invasive
In vivo angiogenesis	Less angiogenic	Less angiogenic	Angiogenic	Highly angiogenic
necrosis	Focal, palisading cells	Focal, palisading cells	Extensive, extravasating blood cells	Mild
Proliferation (Ki67 staining)	Low	Low	high	Low

**Table 3:** Primary antibodies list.

Antigen (Clone/Code)	Source	Antibody
GLAST	Abcam	Rabbit
Nestin	Cell Signaling	Mouse
Olig2	Abcam	Rabbit
Sox2	Sigma-Aldrich	Rabbit
PDGFRa	Santa Cruz	Rabbit
S100b	Abcam	Rabbit
GFAP	Cell Signaling	Rabbit
TuJ1	Sigma-Aldrich	Rabbit
PSA-NCAM	Sigma-Aldrich	Mouse
O4	Sigma-Aldrich	Mouse
NG2	Abcam	Rabbit
YKL-40	Cell Signaling	Rabbit
A2B5	Abcam	Mouse
CD133	Abcam	Rabbit
Ki67	Cell Signaling	Rabbit
vWF	Abcam	Rabbit

## Supplementary Materials

The following are available online at https://www.mdpi.com/2072-6694/11/10/1564/s1, Table S1: Background information on GICs, Table S2: Summary of GIC subtypes differentiation potential in vitro. GIC subtypes cultured under differentiation condition (1% FBS + 1 µM RA) exhibited differential potential of differentiation into neural lineage.
