# Supplementary material for: Genetic and Lineage Classification of Glioma-Initiating Cells Identifies a Clinically Relevant Glioblastoma Model
Source: Cancers (Basel). 2019 Oct 15;11(10):1564. doi: 10.3390/cancers11101564 (PMC6826962; doi:10.3390/cancers11101564)
Supplement: Supplementary file 1 [file cancers-11-01564-s001.pdf]

Supplementary Materials: Genetic and Lineage Classification of Glioma-Initiating Cells Identifies a Clinically Relevant Glioblastoma Model

Norihiko Saito, Nozomi Hirai, Kazuya Aoki, Sho Sato, Ryo Suzuki, Yu Hiramoto, Satoshi Fujita, Haruo Nakayama, Morito Hayashi, Takatoshi Sakurai and Satoshi Iwabuchi

Table S1. Background information on GICs.

Cell line name	Age	Gender	Pathology	Subtype of original tumor	Subtype of GICs
GICT14	62	F	GBM	Neural	Neural
GICT12	48	F	GBM	Neural	Neural
GICT46	75	M	GBM	Neural	Neural
GICT20	55	F	GBM	Neural	Neural
GICT24	58	M	GBM	Neural	Neural
GICT35	56	M	GBM	Neural	Neural
GICT11	51	F	GBM	Proneural	Proneural
GICT22	71	M	GBM	Proneural	Proneural
GICT14	42	M	GBM	Proneural	Proneural
GICT25	63	F	GBM	Proneural	Proneural
GICT18	59	F	GBM	Proneural	Proneural
GICT28	73	F	GBM	Proneural	Proneural
GICT15	64	M	GBM	Classical	Classical
GICT12	38	M	GBM	Classical	Classical
GICT32	66	M	GBM	Classical	Classical
GICT23	52	F	GBM	Classical	Classical
GICT40	69	F	GBM	Classical	Classical
GICT19	47	M	GBM	Classical	Classical
GICT17	68	M	GBM	Mesenchymal	Mesenchymal
GICT38	57	M	GBM	Mesenchymal	Mesenchymal
GICT29	42	F	GBM	Mesenchymal	Mesenchymal
GICT44	65	F	GBM	Mesenchymal	Mesenchymal
GICT22	58	M	GBM	Mesenchymal	Mesenchymal
GICT16	67	M	GBM	Mesenchymal	Mesenchymal
GICT21	55	F	GBM	Mesenchymal	Mesenchymal

Table S2. Summary of GIC subtypes differentiation potential in vitro. GIC subtypes cultured under differentiation condition (1% FBS + 1 uM RA) exhibited differential potential of differentiation into neural lineage.

	Lineage markers	Neural (n = 6)	Proneural (n = 6)	Classical (n = 6)	Mesenchymal (n = 7)
Neural Lineage	Astrocytic (GFAP, S100β)	5/6	6/6	6/6	2/7
	Neuronal (TuJ1)	6/6	6/6	5/6	0/7
	Oligodendrocytic (CNPase, O4)	1/6	6/6	4/6	0/7

© 2019 by the authors. Licensee MDPI, Basel, Switzerland. This article is an open access article distributed under the terms and conditions of the Creative Commons Attribution (CC BY) license (http://creativecommons.org/licenses/by/4.0/).
